# Machine Learning-Enabled Intelligent Analysis of Surface-Enhanced Raman Scattering: Methods, Applications, and Perspectives

**DOI:** 10.3390/molecules31101599

**Published:** 2026-05-10

**Authors:** Zixing Li, Yu Wang, Zi Deng, Jingjing Zhao

**Affiliations:** 1 School of Exercise and Health, Shanghai University of Sport, Shanghai 200438, China; lizixing1026@163.com (Z.L.); wyy202423@163.com (Y.W.); 2 School of Intelligent Sports Engineering, Shanghai University of Sport, Shanghai 200438, China; larrynn2024@163.com; 3 Shanghai Institute of Doping Analyses, Shanghai University of Sport, Shanghai 200438, China

**Keywords:** surface-enhanced Raman scattering (SERS), machine learning (ML), trace analysis, biomarker discovery, nano-substrate optimization

## Abstract

Surface-enhanced Raman spectroscopy (SERS) enables ultrasensitive molecular detection but produces high-dimensional and substrate-dependent spectral data that are difficult to analyze using conventional methods. The integration of machine learning (ML) provides new opportunities for extracting chemical information from complex SERS datasets and for optimizing nanostructured substrates that determine signal enhancement. This review summarizes recent advances in ML-assisted SERS across the analytical workflow. Data characteristics and preprocessing strategies are first outlined, followed by an overview of supervised, unsupervised, and deep learning approaches for spectral classification and quantitative analysis. Applications in biomarker discovery and spectral fingerprint recognition are discussed, with emphasis on model interpretability. In addition, ML-driven strategies for substrate optimization, including surrogate modeling and inverse design, are highlighted as emerging directions for improving enhancement efficiency. Current challenges, such as data scarcity, limited generalization, and real-time deployment constraints, are also examined. The convergence of ML and SERS is gradually shifting Raman-based analysis toward more predictive and integrated sensing frameworks.

## 1. Introduction

Surface-enhanced Raman scattering (SERS) is an ultrasensitive vibrational spectroscopic technique that utilizes the surface plasmon resonance (SPR) properties of metallic nanostructures to amplify Raman signals [1]. In typical SERS measurements, analyte molecules are adsorbed onto or located near nanostructured substrates, commonly composed of silver or gold nanoparticles (AgNPs/AuNPs). Upon laser excitation, the localized surface plasmon resonance (LSPR) generates highly intensified electromagnetic fields around the nanostructures, significantly enhancing the Raman scattering of nearby molecules [2]. The enhancement mechanism is generally attributed to two major contributions: electromagnetic (EM) enhancement and chemical (CM) enhancement. EM enhancement, arising from collective oscillations of conduction electrons, produces strong local fields at nanogaps or sharp features (“hot spots”) and dominates signal amplification [3]. CM enhancement involves interfacial charge transfer or chemical interactions between the analyte molecule and the substrate, providing an additional but typically smaller contribution [4,5]. Due to its ultrahigh sensitivity, molecular fingerprint specificity, and rapid, label-free detection capability, SERS has been widely applied in biomedical diagnostics [6], therapeutic drug monitoring [7], environmental analysis [8], food safety [9], and materials science [10]. In recent years, its potential has further expanded to emerging fields such as illicit drug detection [11] and sports doping control [12], where rapid and reliable trace-level analysis in complex matrices is required.

Despite its significant analytical advantages, the practical application of SERS faces several persistent challenges. First, variations in nanostructure morphology, interparticle spacing, and surface chemistry during substrate fabrication often result in poor signal reproducibility, largely due to the stochastic distribution of electromagnetic “hot spots.” Second, complex sample matrices—particularly in biological or environmental samples—introduce background interference, fluorescence effects, and nonspecific adsorption, which complicate spectral interpretation. In addition, SERS spectra are inherently high-dimensional and frequently contaminated by noise, baseline drift, and instrumental fluctuations. The relationship between spectral intensity and analyte concentration may also exhibit nonlinear behavior due to hotspot saturation or heterogeneous adsorption effects. These factors make reliable feature extraction and quantitative modeling challenging. Conventional analysis strategies, including manual peak assignment, univariate calibration, and traditional multivariate statistical approaches, depend strongly on expert-selected features and predefined assumptions, limiting their robustness and scalability in high-throughput or non-targeted analytical scenarios [13]. Furthermore, optimization of SERS substrate performance often involves multidimensional parameter tuning and empirical trial-and-error processes, which can be time-consuming and inefficient.

Machine learning (ML) has emerged as a powerful data-driven framework to address these analytical challenges. By extracting informative patterns from high-dimensional spectral datasets, ML algorithms enable robust classification and quantitative regression without relying solely on manually selected spectral features [14]. In SERS applications, ML-assisted approaches have been widely employed for spectral denoising, baseline correction, and feature selection, thereby improving analytical reproducibility and model stability. Supervised learning methods such as support vector machines, random forests, and artificial neural networks have demonstrated strong performance in qualitative identification and quantitative analysis of target molecules. Unsupervised learning techniques, including principal component analysis and clustering algorithms, have been used for exploratory data analysis and sample discrimination. More recently, deep learning models—particularly convolutional neural networks—have shown the capability to learn hierarchical representations directly from raw spectral data, reducing dependence on manual feature engineering and enhancing performance in complex datasets. In addition to molecular identification, ML strategies are increasingly being explored for non-targeted spectral pattern recognition and potential biomarker identification in biomedical SERS studies. Furthermore, emerging studies have applied ML to guide SERS substrate design and structural optimization, aiming to accelerate nanomaterial development and improve reproducibility.

Recently, the integration of machine learning and SERS has garnered significant attention, with several excellent reviews published between 2022 and 2025 [15,16,17,18,19,20]. These reviews have significantly advanced the understanding of ML-assisted SERS applications in areas such as biomedical diagnostics, environmental monitoring, and food safety. However, most existing reviews primarily emphasize downstream applications or predictive performance, while providing comparatively limited discussion of the methodological trade-offs among different ML strategies and their suitability for specific SERS analytical challenges. In particular, there remains a need for more critical evaluation of how different ML approaches perform in the context of characteristic SERS data features, including high dimensionality, spectral noise, nonlinear signal variation, and substrate-dependent variability. Furthermore, the connection between data-driven ML models and the underlying physicochemical origins of SERS signals—such as electromagnetic and chemical enhancement mechanisms—has not been comprehensively discussed.

**Figure 1:**
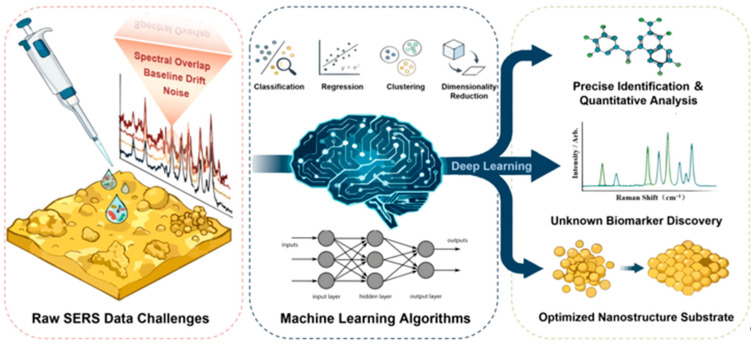
Overview of Machine Learning-Driven SERS Intelligent Analysis.

To bridge these remaining gaps, this review provides an integrated analytical perspective on ML-assisted SERS analysis across the complete analytical workflow (Figure 1). Distinct from the previous literature that mainly focuses on specific applications or isolated algorithms, this review emphasizes: (i) critically comparing algorithm selection tailored to SERS-specific challenges (e.g., spectral noise, high dimensionality, and substrate variability); (ii) deeply integrating physical mechanisms with computational approaches, highlighting emerging trends such as the ML-guided inverse design of nanostructured substrates and the compatibility of novel 2D materials. Through this perspective, we aim to highlight the transition of ML in SERS from a purely post-processing tool toward a more integrated framework that combines intelligent spectral analysis with physically informed substrate and sensing design.

## 2. SERS Data Characteristics and Analytical Challenges

SERS spectral data exhibit distinctive characteristics that differentiate them from conventional vibrational spectra. Through plasmonic enhancement, Raman scattering signals can be amplified by several orders of magnitude, enabling trace-level and even single-molecule detection under optimized conditions. This extraordinary sensitivity provides rich molecular fingerprint information. However, the same physicochemical mechanisms that enable signal enhancement also introduce analytical complexities. Variability in nanostructure-dependent enhancement, spectral interference, and high-dimensional data structures collectively pose significant challenges for reliable quantitative modeling and pattern recognition [2]. To clearly demonstrate the key features of SERS data and the main analytical difficulties in practical detection, these points are outlined in Table 1.

### 2.1. Typical Characteristics of SERS Spectra

SERS spectra are inherently high-dimensional, as each measurement typically contains hundreds to thousands of Raman shift variables across a broad spectral range. This high dimensionality increases computational complexity and may lead to the so-called “curse of dimensionality,” where redundant or collinear variables reduce model generalizability and increase the risk of overfitting.

In addition to dimensional complexity, SERS spectra are frequently affected by multiple sources of noise and interference. High-frequency random noise arises from detector and instrumental fluctuations, while low-frequency background signals—most notably fluorescence from substrates, solvents, or coexisting matrix components—can significantly distort baseline profiles and obscure weak Raman bands [21]. These interferences reduce the effective signal-to-noise ratio and complicate downstream quantitative analysis.

A more fundamental characteristic of SERS is its dependence on substrate nanostructure properties. Signal intensity and spectral stability are strongly influenced by nanoparticle size, morphology, interparticle spacing, aggregation state, and surface chemistry. Because electromagnetic enhancement is concentrated at localized “hot spots,” which are often stochastically distributed, small variations in nanostructure configuration can result in substantial signal fluctuations. This substrate dependence may manifest as batch-to-batch variability, intensity instability, and occasional peak position shifts due to local chemical environment changes or thermal effects [22].

Baseline drift is another common phenomenon, typically originating from fluorescence background, instrumental response variation, or slow changes in environmental conditions. Collectively, high dimensionality, spectral noise, baseline instability, and substrate-dependent variability reduce the direct interpretability of raw SERS spectra. As a result, rigorous data preprocessing and feature engineering are essential prerequisites for reliable statistical analysis and machine learning model development.

### 2.2. Data Preprocessing and Feature Engineering

**Figure 2:**
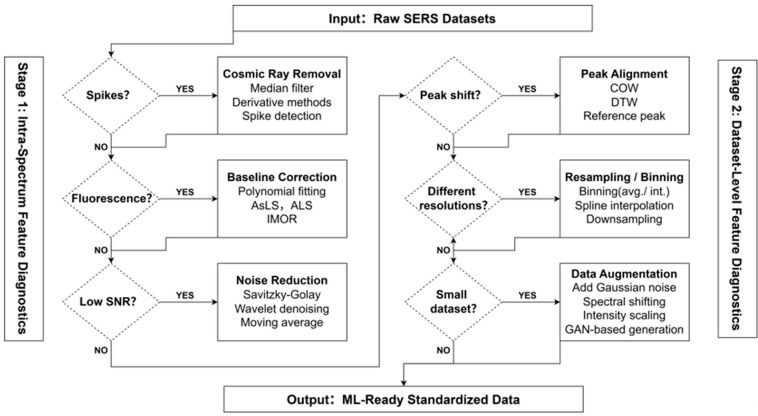
A characteristic-driven decision workflow for SERS data preprocessing. To ensure data integrity for downstream machine learning, the pipeline is divided into two sequential stages.

Systematic data preprocessing transforms raw, noisy SERS spectra into structured and analytically reliable datasets suitable for advanced modeling. Unlike rigid linear pipelines, selecting appropriate preprocessing strategies should be a dynamic process driven by specific data characteristics. As illustrated in Figure 2, a comprehensive preprocessing framework can be logically divided into two sequential stages: intra-spectrum feature diagnostics (Stage 1) and dataset-level feature diagnostics (Stage 2).

#### 2.2.1. Stage 1: Intra-Spectrum Feature Diagnostics

The first stage focuses on correcting internal artifacts within individual spectra.

(i) Cosmic Ray Removal: SERS spectra are typically collected using highly sensitive Charge-Coupled Device (CCD) detectors, which are susceptible to cosmic ray strikes. These manifest as sharp, intense, and narrow positive spikes. If not removed, ML models may erroneously identify these random spikes as significant Raman features. Therefore, applying cosmic ray removal algorithms—such as median filtering or derivative-based methods—is a mandatory first step [23,24,25].
(ii) Baseline Correction: Following spike removal, baseline correction methods are applied to eliminate broad fluorescence backgrounds and instrumental drift. Polynomial fitting [26] and penalized least squares approaches—most notably asymmetric least squares (AsLS) and alternating least squares (ALS) regressions [27,28]—are widely used to estimate and subtract slowly varying baseline components, thereby restoring the true Raman signal profile.
(iii) Noise Reduction/Smoothing: To suppress high-frequency random noise without distorting peak shapes, smoothing techniques such as Savitzky–Golay filtering [29] and wavelet-based denoising [30] are subsequently employed. This step improves the signal-to-noise ratio (SNR), especially for spectra with weak Raman scattering.

#### 2.2.2. Stage 2: Dataset-Level Feature Diagnostics

The second stage addresses inconsistencies across multiple measurements or diverse datasets to ensure uniform input for ML models.

(iv) Peak Alignment: In practical SERS measurements, subtle shifts in Raman peak positions (typically by a few cm^−1^) are frequently observed due to variations in molecular adsorption orientation, thermal effects, or slight instrumental miscalibrations. For ML models that rely on strict wavelength registration, unaligned peaks can be misinterpreted as different chemical species. Spectral alignment techniques like Correlation Optimized Warping (COW) or dynamic time warping (DTW) are essential to align prominent marker bands across the dataset [31,32,33,34].
(v) Spectral Binning and Resampling: When aggregating SERS data from multiple laboratories or different instruments, a major challenge is the discrepancy in spectral resolution and step sizes. ML models require inputs of uniform dimensionality. Spectral binning or spline interpolation-based resampling addresses this by standardizing the number of data points per spectrum [35,36,37].
(vi) Data Augmentation: The implementation of deep learning in SERS is often hindered by ‘data scarcity.’ Acquiring tens of thousands of reproducible SERS spectra experimentally is costly. To prevent models from overfitting on small or imbalanced datasets, techniques such as adding white noise, applying minor spectral shifts, or generating artificial spectra using Generative Adversarial Networks (GANs) are widely employed to robustly expand the training set size [38,39,40,41].

#### 2.2.3. Final Preparation: Normalization and Feature Extraction

Once the data is standardized through the above workflow, intensity normalization is applied to compensate for overall signal variations caused by differences in laser power or substrate heterogeneity. Common approaches include vector normalization, area normalization, and internal standard calibration [35]. Among these, internal standard calibration is theoretically the most ideal strategy, as it effectively corrects for variations in SERS hot-spot distribution and local field enhancement—a unique challenge in SERS analysis. However, it requires careful experimental design, including the selection of appropriate internal standards and validation of their non-interference with target analytes. When such experimental conditions are not met, vector or area normalization offers more practical alternatives.

Finally, dimensionality reduction and feature extraction become critical steps before model training. Direct modeling using all wavenumber variables is often inefficient due to multicollinearity. Multivariate techniques like principal component analysis (PCA) [42] are widely adopted to project spectra into a lower-dimensional latent space. Through this holistic preprocessing and feature engineering procedure, SERS data are converted from unstructured, noise-affected signals into organized, ML-ready representations.

## 3. Machine Learning Methods for SERS Analysis

Building upon the intrinsic characteristics and analytical challenges of SERS data discussed in Section 2, ML provides a structured and data-driven framework for converting complex spectral measurements into reliable analytical models. Rather than relying solely on manual spectral interpretation, ML-based SERS analysis follows a systematic workflow that integrates experimental data generation with computational modeling.

**Figure 3:**
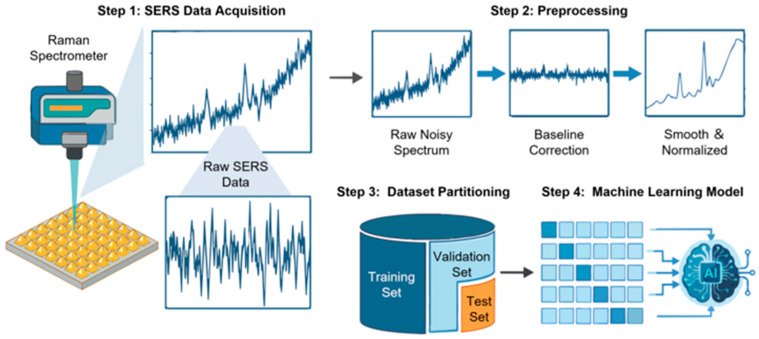
Typical workflow of ML-based SERS data interpretation, including SERS data acquisition, spectral preprocessing, dataset partitioning, and machine learning model development and evaluation.

As illustrated in Figure 3, a typical ML-based SERS data interpretation process consists of four key steps: SERS data acquisition, spectral preprocessing, dataset partitioning, and machine learning model construction and evaluation. This workflow highlights the close coupling between experimental design and computational analysis, emphasizing that model performance is not determined by algorithm selection alone but by the quality and structure of data throughout the entire analytical pipeline.

### 3.1. Typical Process for ML-Based SERS Analysis

#### 3.1.1. SERS Data Acquisition

The workflow begins with SERS data acquisition, where spectral measurements are collected under controlled experimental conditions. The quality, reproducibility, and representativeness of acquired spectra directly influence downstream preprocessing and modeling performance. Factors such as substrate fabrication consistency, laser parameters, spectral resolution, and sampling strategy must be carefully optimized to ensure reliable data generation.

#### 3.1.2. Data Preprocessing

As systematically detailed in Section 2.2, transforming raw SERS spectra into reliable datasets requires a characteristic-driven preprocessing workflow. Within the context of machine learning model development, the execution of these steps must be strictly controlled. The primary focus at this stage is to ensure that the mathematical transformations do not introduce methodological errors. Importantly, to avoid information leakage (data leakage), all preprocessing parameters—such as baseline fitting coefficients, normalization factors, and principal component matrices—must be derived exclusively from the training dataset [43]. These learned parameters are then uniformly applied to the validation and test sets. In addition to the spectral corrections previously discussed, ML-specific preparations often involve feature scaling (e.g., standardization or min-max scaling) and rigorous outlier treatment to stabilize model training, accelerate convergence, and reduce multicollinearity [44]. Ultimately, executing this standardized pipeline ensures that downstream algorithms learn chemically meaningful variations rather than experimental or computational artifacts.

#### 3.1.3. Dataset Partitioning

After preprocessing, the curated dataset must be systematically partitioned to enable reliable model training and unbiased evaluation, ensuring that performance estimates reflect true generalization ability rather than the memorization of training data. Typically, datasets are strictly divided into a training set for parameter learning, a validation set for hyperparameter tuning, and an independent test set for final performance evaluation. In SERS studies where sample sizes are often limited or imbalanced, cross-validation strategies—such as k-fold cross-validation or stratified sampling—are widely adopted to improve robustness and reduce variance in performance estimation. When hyperparameter tuning is involved, nested cross-validation can further minimize bias [45]. Ultimately, transparent reporting of these data partitioning strategies is essential to ensure reproducibility and comparability across studies.

#### 3.1.4. Machine Learning Model Development

Following dataset partitioning, machine learning models are constructed to identify patterns and generate predictive outputs based on the defined analytical objective, such as classification, regression, clustering, or dimensionality reduction. Model development generally includes algorithm selection, hyperparameter optimization, model training, and final evaluation. The selection of appropriate algorithms should be aligned with the specific problem type, data characteristics, and practical considerations such as predictive accuracy and model interpretability [18]. For exploratory analysis and visualization, principal component analysis (PCA) [46] is widely used to reveal variance structures and clustering trends. Supervised classification models such as linear discriminant analysis (LDA) [47] and partial least squares discriminant analysis (PLS-DA) [48] are commonly applied for differentiating sample groups, while more flexible algorithms—including support vector machines (SVM) [49] and random forests (RF) [50]—are often employed for nonlinear and high-dimensional SERS data to enhance classification robustness. In quantitative applications, regression models such as partial least squares regression (PLSR) [51] and kernel-based methods are used to establish relationships between spectral features and analyte concentration. More recently, deep learning architectures, particularly convolutional neural networks (CNNs), have demonstrated the ability to learn hierarchical spectral representations directly from raw or minimally processed SERS data, though their performance typically depends on dataset size, quality, and appropriate regularization strategies [52].

Rigorous performance evaluation is essential to ensure the reliability, generalizability, and practical applicability of these ML models in SERS analysis. The choice of evaluation metrics depends on the task type: classification problems commonly report accuracy, precision, recall, F1-score, and area under the receiver operating characteristic curve (AUC-ROC), with F1-score or AUC being more informative in cases of class imbalance. For regression tasks, performance is typically assessed using mean squared error (MSE), root mean squared error (RMSE), and mean absolute error (MAE) [53]. Importantly, these metrics should be calculated on independent validation or test datasets rather than solely on training data to prevent overfitting. Techniques such as k-fold cross-validation or nested cross-validation are recommended to improve robustness, particularly in studies with limited sample sizes, and transparent reporting of data splitting strategies, preprocessing steps, and hyperparameter settings is critical for reproducibility [54]. Furthermore, ML model construction for SERS analysis is inherently iterative; if model performance is unsatisfactory, adjustments may include revisiting preprocessing strategies, refining feature extraction methods, optimizing hyperparameters, or selecting alternative algorithms. The interplay between data quality, algorithm choice, and validation rigor ultimately determines model reliability.

While Section 3.1 outlines the procedural workflow of ML-based SERS analysis, a deeper understanding of model selection requires distinguishing between different learning paradigms and architectural strategies. The choice of model is not merely a technical step within the workflow; rather, it reflects how spectral information is represented, how supervision is incorporated, and how model complexity is balanced against dataset scale. Therefore, it is necessary to examine the principal categories of machine learning models applied to SERS and to clarify their respective strengths, limitations, and suitable application scenarios.

### 3.2. Learning Paradigms and Model Architectures in SERS Analysis

Building upon the general ML workflow described above, machine learning approaches applied to SERS data can be categorized according to their learning paradigms and model architectures. Rather than viewing these categories as isolated techniques, it is more meaningful to consider how different model types align with specific analytical objectives and data characteristics inherent to SERS spectroscopy.

#### 3.2.1. Traditional Machine Learning Models

Traditional machine learning models remain the cornerstone of SERS analysis, primarily due to their low computational requirements, strong performance on small to moderate-sized datasets, and high chemical interpretability. Rather than broadly categorizing these algorithms into textbook definitions of classification and regression, it is more practically relevant to examine how they address specific SERS data challenges.

For qualitative tasks (e.g., disease diagnosis or chemical species identification), algorithms such as support vector machines (SVM) [49], random forests (RF) [50], decision trees (DT) [55], Naïve Bayes (NB) [56], and K-nearest Neighbors (KNN) [57] are extensively utilized. Notably, chemometric approaches like linear discriminant analysis (LDA) [47] and partial least squares discriminant analysis (PLS-DA) [48] remain highly favored when attributing spectral features to specific chemical bonds is paramount. For quantitative trace analysis, Linear Regression [58], Regression Trees (RT) [59], and partial least squares regression (PLSR) effectively model concentration-dependent spectral variations, especially when signals exhibit approximately linear behaviors.

To manage the inherent high dimensionality of SERS data, unsupervised techniques such as principal component analysis (PCA) [46] and t-distributed Stochastic Neighbor Embedding (t-SNE) [60] serve as essential precursors for redundancy reduction and visualization. Concurrently, K-means [61] and hierarchical clustering [62] are applied to reveal intrinsic grouping in complex spectral matrices. Furthermore, to address the common challenge of limited labeled SERS spectra, semi-supervised strategies—ranging from graph-based label propagation [63] to Generative Adversarial Network (GAN) architectures [64]—are increasingly explored to leverage large volumes of unlabeled spectral data.

#### 3.2.2. Deep Learning Models

Unlike traditional models that rely heavily on handcrafted feature engineering, deep learning (DL) autonomously extracts hierarchical representations directly from raw or minimally processed SERS spectra [65,66].

Common DL architectures have been specifically tailored to SERS characteristics. Artificial neural networks (ANN) [67] and Multi-Layer Perceptron (MLP) [68] are widely used to model complex nonlinear relationships. Convolutional neural networks (CNNs) [69] are uniquely suited for 1D Raman spectra, as their convolutional kernels effectively capture localized peak shapes and subtle shoulder features while resisting high-frequency noise. Recurrent Neural Networks (RNNs), including LSTM and GRU [70], are designed to exploit sequential dependencies across adjacent Raman shifts. More recently, Residual Neural Networks (ResNet) [71,72] have been introduced to extract discriminative features from highly complex matrices without gradient vanishing.

#### 3.2.3. Critical Comparison: Chemometrics vs. Deep Learning

Despite the hype surrounding advanced algorithms, a critical trade-off exists between model interpretability and predictive accuracy. Traditional chemometrics (e.g., PLS-DA) and tree-based models (e.g., RF) offer high transparency, allowing chemists to trace classification rules back to specific vibrational modes (Raman shifts). This physical interpretability is crucial for biological credibility and clinical validation. Conversely, DL architectures operate largely as “black boxes,” posing challenges for mechanistic understanding.

Deep learning does not universally replace traditional chemometrics. Conventional ML algorithms demonstrate superior stability and robustness in small-sample settings typical of early-stage SERS experiments. DL truly outperforms traditional methods only when deployed on large-scale, heterogeneous datasets characterized by severe spectral overlap, nonlinear matrix interference, and intense background noise. Therefore, model selection should not be driven by algorithmic complexity, but rather guided by dataset size, spectral noise, and interpretability requirements. Table 2 provides a practical summary of these algorithms, contrasting their strengths and limitations to guide appropriate model selection based on specific SERS data characteristics.

## 4. The Key Role of Machine Learning in Intelligent SERS Analysis

Owing to its ultrahigh sensitivity and molecular fingerprint specificity, SERS has demonstrated significant potential in biological diagnostics, illicit drug detection, environmental monitoring, and sports doping analysis [73,74]. Depending on analytical objectives, SERS applications can generally be categorized into two modes: non-targeted detection (e.g., biomarker discovery or contaminant screening) and targeted detection (e.g., identification and quantification of predefined analytes) [19].

Despite these advantages, conventional SERS analysis faces several persistent challenges. Targeted detection often relies on specific recognition elements that suffer from environmental instability. In non-targeted detection, spectral overlap and matrix interference complicate reliable identification, particularly when low-abundance compounds are masked by dominant signals. Furthermore, manual spectral interpretation lacks scalability and robustness [20]. Machine learning provides a systematic solution to these limitations. Rather than replacing SERS, ML enhances its analytical robustness, interpretability, and throughput, transforming SERS into a more intelligent sensing platform.

### 4.1. Precise Target Molecule Identification and Quantitative Analysis

In complex biological or forensic samples, strong background signals arise from nonspecific adsorption of matrix components, while target molecules may exist at trace levels. Overlapping Raman peaks often exceed the capability of manual interpretation. Machine learning algorithms effectively address these issues through automated feature selection and multivariate classification. As summarized in Table 3, traditional supervised models like SVM and DA have been successfully deployed for the rapid screening of structurally similar illicit drugs (e.g., amphetamines and fentanyl) directly from complex biofluids by capturing distinct spectral variance [75,76].

**Figure 4:**
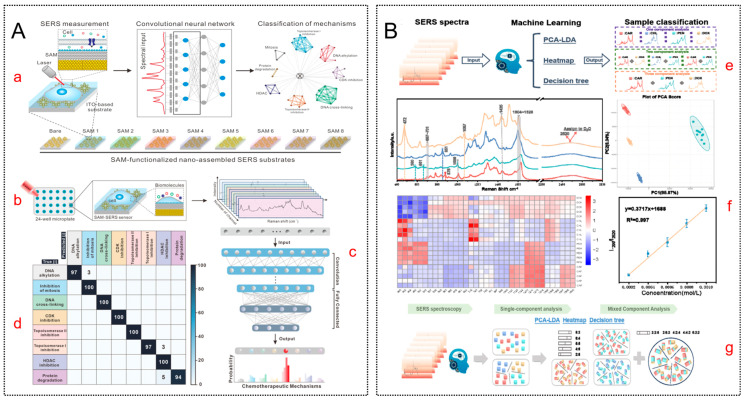
Machine learning-enabled SERS for precise molecular identification and quantitative analysis. (**A**) Convolutional neural network (CNN)-based classification of chemotherapeutic mechanisms using SAM-modulated SERS fingerprints. (**a**) Schematic workflow of the study. (**b**) Experimental setup using BT549 cells. (**c**) Construction of the input data matrix (1022 × 9). (**d**) Model performance evaluated by a confusion matrix, illustrating classification across eight drug mechanisms. Reproduced with permission from Ref. [77]. (**B**) SERS platform integrating dual signal enhancement with machine learning for veterinary drug detection. (**e**) Overall workflow of the ML-based classification framework for different veterinary drugs. (**f**) Quantitative analysis of single-component drugs, including SERS spectra, 2D PCA score plots, thermograms, and calibration curves based on Raman intensity ratios (I_1299_/I_2520_). (**g**) Qualitative analysis of mixed-component drugs, showing the classification workflow together with LDA score plots, heatmaps, and decision tree models. Reproduced with permission from Ref. [79].

Deep learning approaches further enhance the discrimination of highly overlapping compounds. For instance, converting 1D spectra into a 2D spectral matrix as CNNs input allows the model to learn hierarchical representations and preserve inter-channel correlations. As detailed in Figure 4A, this approach yielded high-precision classification of chemotherapeutic mechanisms across 20 drugs, significantly outperforming conventional 1D input methods [77].

Beyond qualitative classification, quantitative SERS analysis requires establishing reliable mapping relationships between spectral features and analyte concentrations. While traditional linear calibration models (e.g., PLSR) perform well in simple systems, they often struggle in interference-rich matrices. Here, nonlinear and ensemble ML algorithms significantly improve quantification. For example, RF has been utilized for trace sedative detection; importantly, RF provides feature importance analysis, allowing researchers to trace predictions back to chemically meaningful Raman stretching regions (e.g., C–Br bonds), thereby enhancing model interpretability [78].

To achieve comprehensive sample profiling, multimodal hybrid ML platforms are increasingly adopted. By combining PCA-LDA for classification, decision trees for rule extraction, and Multivariate Curve Resolution—Asymmetric least squares (MCR-ALS) for quantitative resolution, researchers have achieved 100% accuracy in profiling mixed veterinary drugs (Figure 4B) [79]. Furthermore, in applications demanding rapid, automated screening like sports doping detection, sequence-aware deep learning models such as LSTMs excel by capturing long-range dependencies and sequential correlations within spectral data, demonstrating superior robustness over traditional classifiers [80].

Collectively, these studies illustrate that the integration of machine learning with SERS does not merely improve classification accuracy; it enhances robustness against matrix interference, enables nonlinear quantitative modeling, and facilitates the automation of spectral interpretation.

### 4.2. Identification and Discovery of Biomarkers for Unknown Molecules

Unlike targeted detection, early-stage biomarker discovery in complex biological systems aims to identify spectral patterns associated with pathological states without prior specification of molecular targets. In Raman spectroscopy, biomarkers often manifest not as single isolated peaks, but as coordinated spectral variations corresponding to biochemical alterations. Conventional reductionist approaches focusing on individual molecules often suffer from limited predictive power and poor reproducibility. Integrating SERS with machine learning shifts the paradigm toward data-driven spectral analysis. As summarized in Table 3, algorithmic regression models are instrumental in validating the presence of low-abundance biomarkers (e.g., circulating miRNAs), bridging the gap between spectral findings and clinical diagnostics [81].

**Figure 5:**
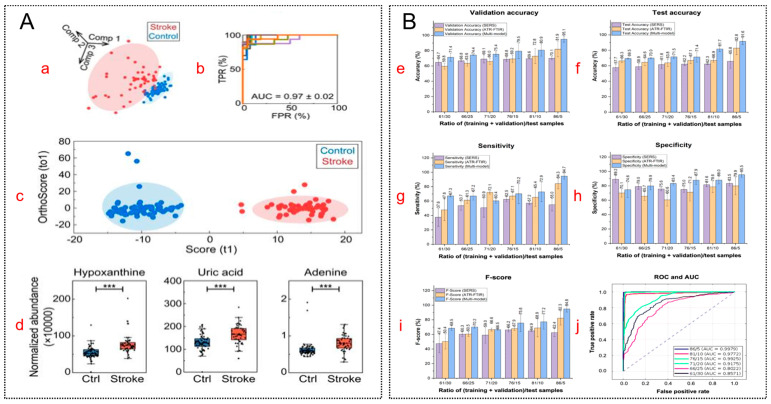
Machine learning-assisted identification and discovery of biomarkers from SERS and multimodal spectral data. (**A**) Biomarker screening for ischemic stroke. (**a**) Visualization of mean spectral differences using sPLS-DA. (**b**) Classification performance evaluated by ROC curves of SVM-LDA models over 10 repeated runs (mean AUC = 0.97 ± 0.02). (**c**) OPLS-DA score plots differentiating controls and patients with ischemic stroke based on HPLC–MS/MS data (positive ion mode). (**d**) Box plots showing normalized abundances of key biomarkers (hypoxanthine, uric acid, and adenine) in stroke versus control groups. Biomarker selection criteria include ANOVA significance, fold change > 1.3, VIP > 1, and CV < 30%. *** *p* < 0.001 vs. the control group. Reproduced with permission from Ref. [82]. (**B**) Performance comparison of machine learning models under different data modalities and training/test splits. (**e**–**i**) Evaluation metrics, including validation accuracy, test accuracy, sensitivity, specificity, and F-score. Color coding indicates data modality: SERS (purple), ATR-FTIR (orange), and multimodal fusion (blue). Bar values represent means from three independent runs, with error bars indicating standard deviation. (**j**) ROC curves and corresponding AUC values demonstrating improved performance of the multimodal fusion strategy. Reproduced with permission from Ref. [83].

Machine learning enables the efficient processing of complex metabolomic data by exploiting global spectral patterns to capture subtle biochemical changes. For example, combining sparse PLS discriminant analysis (sPLS-DA) for dimensionality reduction with SVM classification not only accurately distinguished patients with stroke from healthy controls but also successfully identified hypoxanthine as a differential metabolite (Figure 5A) [82]. This illustrates a critical methodological shift: biomarker discovery is no longer restricted to identifying isolated molecules, but instead focuses on discovering discriminative signatures embedded within complex metabolic networks. Furthermore, diagnostic discrimination can be synergistically enhanced through multimodal fusion. As shown in Figure 5B, integrating attenuated total reflectance—Fourier transform infrared spectroscopy (ATR-FTIR) with SERS via a unified ML pipeline significantly outperformed single-modality approaches for breast cancer detection [83].

To manage complex clinical samples, coupling SERS with microfluidic platforms allows ML models (e.g., PLS-DA) to integrate multiple spectral features from different exosomal surface proteins into a unified diagnostic framework, vastly improving robustness over single-marker strategies [84].

Despite the strong predictive performance of advanced ML architectures, concerns regarding interpretability remain. In clinical contexts, understanding which spectral features drive classification decisions is crucial for biological credibility and regulatory acceptance. Recent efforts have thus pivoted toward Explainable Artificial Intelligence (XAI) applied to SERS. For instance, Cheng et al. [85] constructed a deep neural network (DNN)-based “digital retina” model utilizing Score-weighted visual explanations for convolutional neural networks (Score-CAM) visualization to analyze serum SERS spectra. The model automatically identified discriminative spectral peaks as candidate “digital biomarkers.” This represents an important conceptual advancement: instead of relying on predefined markers, ML-assisted SERS enables panoramic spectral fingerprint discovery while retaining interpretability through feature attribution. Such approaches effectively transition SERS diagnostics from opaque “black-box” classifications toward transparent and biologically meaningful decision-making frameworks.

Collectively, these studies demonstrate that machine learning plays multiple roles in biomarker discovery: extracting discriminative spectral signatures from high-dimensional data, integrating multimodal biochemical information, and enabling interpretable feature discovery through XAI techniques. By leveraging global spectral patterns, ML-assisted SERS analysis provides a systems-level perspective on disease-associated biochemical alterations, significantly expanding its potential for early disease diagnosis.

### 4.3. Data-Driven Optimization of SERS Nanostructured Substrates

High-sensitivity SERS detection critically depends on substrate design and optimization of enhancement mechanisms. Electromagnetic (EM) and chemical (CM) enhancement effects are strongly influenced by nanostructure geometry, composition, and spatial arrangement [86]. Conventional substrate development has largely relied on empirical synthesis strategies, in which nanoparticle size, morphology, and material composition are tuned through iterative experimentation. While this trial-and-error approach has led to numerous high-performance substrates, it often lacks systematic predictability and design efficiency. Machine learning introduces a paradigm shift by enabling data-driven prediction and inverse design of nanostructured substrates, thereby reducing reliance on purely empirical exploration [87].

Topology optimization and computational electromagnetic modeling have long been employed to enhance SERS performance. By solving Maxwell’s equations [88], researchers can predict optical responses and local field distributions of candidate nanostructures under defined physical constraints. For example, Yao et al. [89] applied density-based topology optimization combined with an adjoint gradient method to design periodic metal–fluid interface nanostructures. Their approach achieved an average fourfold enhancement compared to conventional spherical or bowtie structures, even under random molecular distributions. Although such physics-driven optimization strategies provide valuable insight, they remain computationally intensive. Full-wave simulations (e.g., Finite-Difference Time-Domain, FDTD) for each candidate nanostructure may require hours of computation, significantly limiting large-scale parameter exploration. This computational bottleneck motivates the integration of machine learning to accelerate optical response prediction and enable inverse design [90].

**Figure 6:**
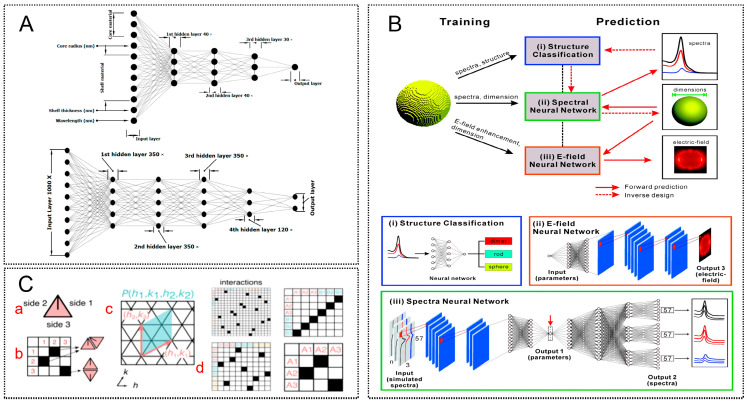
Machine learning-assisted design and optimization of SERS nanostructured substrates. (**A**) Neural network architectures for forward prediction and inverse design of plasmonic core–shell nanostructures. The absorption spectra are predicted using an absorption prediction network (APN), while structural parameters are retrieved via an inverse design network-Regressor (IDN-Regressor). Reproduced with permission from Ref. [92]. (**B**) Deep learning framework for predicting optical responses and guiding nanoparticle design. Three models are trained on FDTD simulation data: (**i**) a structure classification model, (**ii**) a spectral DNN for far-field response prediction, and (iii) an electric-field (E-field) DNN for near-field enhancement mapping. Solid and dashed red arrows represent forward prediction and inverse design pathways, respectively. Reproduced with permission from Ref. [93]. (**C**) Symmetry-guided inverse design strategy for constructing 2D plasmonic assemblies. (**a**) Triangular building block used as the fundamental unit. (**b**) Interaction matrix defining binding rules between components. (**c**) Generation of periodic unit (PU) cells through vector combination. (**d**) Reconstruction of interaction matrices from the resulting tiling patterns. Reproduced with permission from Ref. [94].

Artificial neural networks and related models have been increasingly adopted to learn nonlinear mappings between geometric parameters (e.g., particle size, morphology, and arrangement) and optical responses (e.g., absorption spectra and near-field enhancement distributions). Wang et al. [91] proposed a backpropagation (BP) neural network for inverse prediction of gold nanosphere radius and surrounding refractive index directly from localized surface plasmon resonance (LSPR) spectra. Compared with conventional least-squares fitting, the neural network demonstrated improved flexibility and reduced prediction error. To overcome the high computational cost of iterative simulations, deep learning-based surrogate models have emerged as powerful alternatives. As illustrated in Figure 6, recent studies have successfully deployed ANNs to predict optical responses with high fidelity. For instance, Vahidzadeh et al. [92] (Figure 6A) introduced specific architectures such as the APN and IDN-Regressor to accurately map the relationship between core–shell geometries and their absorption spectra. Similarly, He et al. [93] (Figure 6B) extended this capability by training distinct deep neural networks (DNNs) to predict both far-field spectra and near-field electromagnetic enhancement distributions. Notably, the latter study reported an acceleration of up to six orders of magnitude relative to traditional FDTD simulations. These results demonstrate that, once trained, such ML frameworks can effectively replace computationally expensive solvers, enabling the rapid exploration of complex nanostructures.

This computational efficiency lays the groundwork for inverse design, where the desired optical response dictates the structural parameters. While He et al. utilized deep neural networks to map spectra back to dimensions, Hayakawa et al. [94] (Figure 6C) advanced this concept by incorporating fabrication constraints. They introduced a symmetry-based inverse design framework that uses wallpaper group symmetries to guide the DNA origami assembly of gold nanoparticle superlattices. This method successfully bridged the gap between algorithmic design and physical realization, enabling the controlled fabrication of periodic structures (up to 300 nm periodicity) with tunable plasmonic responses. Rahman et al. [95] employed a conditional variational autoencoder (cVAE) to generate diverse core–shell nanoparticle geometries under identical target spectral conditions. Compared with sequential neural networks, the cVAE approach improved robustness, structural diversity, and prediction accuracy. These developments mark a transition from parameter tuning toward true inverse engineering of SERS substrates, where machine learning acts as a bridge between target optical functionality and nanostructure geometry.

Beyond classical electromagnetic (EM) frameworks that rely predominantly on noble metal plasmonics, recent advancements have highlighted the immense potential of emerging two-dimensional (2D) materials and non-plasmonic substrates. These novel systems—such as transition metal dichalcogenides (TMDCs) and MXenes—operate primarily through chemical mechanism (CM) and excitonic enhancement [96]. Incorporating these advanced materials aligns perfectly with the new trends of ML applications in SERS. Traditional metallic “hot spots” are inherently stochastic, causing significant batch-to-batch variations that often confound ML algorithms and induce severe “batch effects.” In contrast, 2D materials offer highly uniform atomic surfaces and predictable charge-transfer interactions, resulting in superior spectral reproducibility [97].

This intrinsic structural uniformity is highly compatible with data-driven models, substantially reducing the burden of complex spectral preprocessing. Furthermore, ML is increasingly being employed to decipher these non-classical enhancement mechanisms. By integrating ML with quantum mechanical calculations and molecular dynamics, researchers can now predict non-plasmonic and excitonic enhancement factors, mapping out charge-transfer pathways. Such pathways are often difficult to isolate experimentally using conventional approaches. This deep integration of ML with emerging 2D materials signifies a paradigm shift from purely EM-based optimization to the comprehensive, data-driven design of hybrid, CM-dominated SERS platforms.

In summary, ML-assisted substrate optimization shifts SERS material development from empirical iteration to predictive and generative design. The integration of physics-based simulation, neural-network surrogate modeling, inverse generative frameworks, and emerging 2D materials enables the accelerated exploration of high-dimensional parameter spaces and the systematic design of nanostructures tailored to specific sensing tasks. Such data-driven material engineering complements ML-based spectral analysis, forming a closed-loop intelligent SERS system in which both signal generation and signal interpretation are computationally optimized.

### 4.4. Methodological Synthesis: Toward Intelligent and Integrated SERS Systems

Across Section 4.1, Section 4.2 and Section 4.3, machine learning emerges as a unifying methodological framework in SERS research, simultaneously enabling advanced spectral interpretation and quantitative modeling, facilitating biomarker discovery through high-dimensional pattern recognition, and supporting substrate optimization via predictive and inverse nanostructure design. By integrating these analytical and material-engineering dimensions, ML extends its role beyond post-acquisition data processing to actively influence experimental strategy, substrate fabrication, and overall system performance.

This convergence effectively redefines SERS as an intelligent and integrated sensing platform rather than a standalone spectroscopic technique. The synergy between ML-driven spectral analytics and ML-assisted substrate engineering establishes the conceptual foundation for closed-loop SERS systems, in which spectral feedback iteratively guides substrate redesign, and optimized nanostructures, in turn, generate higher-quality data that enhance subsequent model training and refinement.

Realizing such an adaptive framework, however, requires standardized datasets, reproducible fabrication protocols, and transparent validation strategies to ensure that computational optimization translates into practical analytical reliability. In this context, the deep integration of machine learning with SERS represents a methodological evolution—from empirical signal enhancement and manual interpretation toward predictive, adaptive, and system-level intelligent sensing capable of real-world deployment.

## 5. Fundamental Challenges and Future Perspectives

Although machine learning and deep learning have substantially advanced SERS data analysis, their widespread and reliable deployment remains constrained by several fundamental challenges. Among these, the most critical limitation is data availability and standardization. The performance of most machine learning algorithms depends heavily on large-scale, high-quality annotated datasets [98]; however, generating reproducible SERS spectral data at such scale is inherently difficult. The stochastic distribution of electromagnetic “hot spots,” variations in nanostructured substrate morphology, charge-transfer interactions between analytes and metallic surfaces, and environmental fluctuations collectively introduce significant signal variability. These intrinsic physicochemical complexities not only increase experimental cost but also hinder cross-platform reproducibility and database standardization. Consequently, data scarcity and heterogeneity remain major bottlenecks restricting model robustness and generalization.

To overcome this bottleneck, a critical paradigm shift toward rigorous data standardization is imperative. Currently, the lack of unified reporting standards prevents the effective merging of datasets from different laboratories. The SERS community must actively move towards establishing open-source, large-scale, standardized spectral databases (analogous to ‘ImageNet’ in computer vision). Crucially, these repositories must enforce strict metadata reporting protocols—mandating detailed records of substrate morphology, laser wavelength, integration time, and sample matrix conditions. Only through such systematic standardization can SERS data become truly reusable, enabling the training of foundational ML models. Such standardized databases would also provide the ideal source domain for training transferable models, enabling effective domain adaptation to target-specific instruments or conditions.

Beyond data limitations, achieving reliable model generalization remains a profound challenge for practical deployment. In SERS analysis, a deep learning model trained on spectra from one specific Raman spectrometer or a single batch of substrates frequently experiences drastic performance degradation when applied to different instrumental setups or novel biological matrices. This ‘domain shift’ highlights that models often learn batch-specific artifacts rather than universal chemical rules. To achieve true generalizability, future research must move beyond simply training models from scratch on isolated datasets. Instead, it is essential to incorporate advanced techniques such as transfer learning and domain adaptation, which can recalibrate and align spectral features across disparate instruments and substrate batches. By doing so, models can retain their diagnostic accuracy even when deployed in diverse, unseen real-world clinical or field environments. This performance instability reflects a deeper methodological concern: many existing models primarily capture statistical correlations rather than physically meaningful relationships. As a result, they frequently function as “black boxes,” offering limited mechanistic insight into the molecular–substrate interactions that fundamentally govern SERS enhancement. For an analytical technique rooted in physicochemical principles, such opacity constrains both scientific understanding and practical trustworthiness.

Another practical concern arises from the mismatch between computational complexity and real-time analytical demands. Advanced deep learning architectures require substantial computational resources for training and inference, which may conflict with the need for portable, rapid, on-site SERS diagnostics. Bridging this gap, therefore, necessitates not only algorithmic innovation but also hardware–software co-design, crucial for enabling intelligent models to operate efficiently within compact sensing platforms. To achieve “end-to-end” immediate diagnostics and intelligent monitoring, future integrated SERS analysis systems will likely incorporate elements such as low-power Central Processing Units (CPUs), portable SERS detecting chips, lightweight AI models, and small spectrometers [99].

Addressing these challenges will require a conceptual transition from purely data-driven modeling toward physics-integrated intelligent systems. Rather than relying exclusively on large annotated datasets, future machine learning frameworks should incorporate intrinsic SERS enhancement mechanisms—such as electromagnetic field distribution and charge-transfer dynamics—directly into the modeling process. This integration, exemplified by approaches like Physical Information Neural Networks (PINNs) [100] or generative models that synthesize training data embedded with complex physical rules, can significantly enhance model generalization, dependability, and interpretability, particularly under sparse or constrained data conditions. Complementing these strategies, self-supervised learning methods capable of extracting universal representations from unlabeled spectral datasets offer a promising solution to the issue of annotation scarcity.

The integration of multimodal information is also likely to play a transformative role. By jointly analyzing SERS spectra alongside complementary data—such as substrate morphology images, molecular dynamics simulations, or fabrication parameters—machine learning models can more accurately capture intricate structure–property relationships. For instance, simultaneously correlating SERS spectra with related substrate morphology images [101] allows models to establish more precise connections between structural features and observed spectral properties. This multimodal fusion directly supports the previously discussed closed-loop design framework, facilitating the synergistic evolution of substrate optimization and spectral interpretation rather than their independent development.

Looking forward, the ultimate evolution of intelligent SERS systems lies in the establishment of a fully integrated feedback architecture. In this envisioned paradigm, machine learning not only interprets spectral signals but also guides substrate design, experimental parameter selection, and adaptive optimization. High-quality spectral data refine predictive models; improved models inform nanostructure engineering; and optimized substrates generate more stable and information-rich spectra. Signal generation and signal interpretation thus become dynamically coupled components of a unified analytical ecosystem.

Nevertheless, it must be acknowledged that the ultimate performance ceiling of SERS remains bounded by intrinsic physicochemical constraints, including stochastic adsorption behavior and nanoscale electromagnetic variability. Overcoming these limits will require interdisciplinary collaboration spanning nanophotonics, analytical chemistry, artificial intelligence, materials science, and biomedical engineering.

In conclusion, the integration of machine learning with SERS does not merely enhance data processing efficiency; it redefines the methodological foundation of the technique. The field is transitioning from empirical enhancement and post hoc statistical classification toward predictive, interpretable, and adaptive intelligent sensing systems. Achieving this transformation will depend on standardized datasets, physics-aware modeling strategies, multimodal integration, and deployable hardware platforms. Through such coordinated advances, SERS is poised to evolve into a next-generation intelligent analytical technology capable of robust real-world implementation.

## Figures and Tables

**Table 1:** Key characteristics of SERS data and associated analytical challenges.

Feature	Description	Analytical Challenge
High dim. * & Data scarcity	10^2^–10^3^ Raman shifts per spectrum with limited sample sizes.	Overfitting risk; requires dimensionality reduction & data augmentation.
Noise & Backgrounds	Cosmic ray spikes, high-frequency noise, and matrix fluorescence.	Demands rigorous spike removal, denoising, and baseline correction.
Substrate dependence	Signals vary with nanoparticle morphology and stochastic hotspots.	Poor inter-batch reproducibility; necessitates strict intensity normalization.
Peak position variation	Raman shifts vary due to chemical interactions or instrument calibration.	Requires peak alignment to avoid species misclassification.
Intensity instability	Signal fluctuations from laser variations or differing spectral resolutions.	Requires spectral resampling/binning and uniform standardization.

* Dimensionality.

**Table 2:** Summary of machine learning algorithms, their applications, strengths, and limitations.

Algorithm	Task	Strengths	Limitations	Refs.
**Supervised learning models**				
LDA	Class. & Dim.Red. ^1^	Maximizes class separability; fast analytic solution.	Requires prior PCA for SERS (variables > samples); linear boundaries.	[47]
PLS-DA/PLS	Classification	Handles collinear Raman shifts; chemometrics gold standard.	Fails with severe nonlinear matrix effects and baseline drift.	[48]
SVM	Classification	Handles collinear Raman shifts; chemometrics gold standard.	Fails with severe nonlinear matrix effects and baseline drift.	[49]
RF	Class. & Reg. ^2^	Extracts feature importance; highly noise-resistant.	Less interpretable than simple trees; slower prediction.	[50]
DT/CART	Class. & Reg. ^2^	Highly interpretable; maps rules to specific Raman peaks.	Unstable and highly prone to overfitting on noisy SERS data.	[55]
Naïve Bayes	Classification	Extremely fast training for simple mixture screening.	Fails when adjacent Raman peaks are highly correlated.	[56]
KNN	Classification	Simple baseline method for direct spectral matching.	Highly sensitive to SERS intensity fluctuations.	[57]
Linear Regression	Regression	Simple baseline for quantitative trace analysis.	Fails under “hot spot” saturation and nonlinear adsorption.	[58]
XGBoost	Class. & Reg. ^2^	Handles complex spectral overlaps with high accuracy.	Prone to fitting instrumental noise if poorly tuned.	[65]
ANN/MLP	Class. & Reg. ^2^	Captures complex nonlinear concentration–intensity relationships.	“Black box” lacking interpretability; requires large datasets.	[67,68]
CNN/ResNet	Classification	Learns peak shapes and shoulders directly from raw data.	“Black box”; prone to overfitting due to SERS data scarcity.	[69,71]
RNN/LSTM	Regression	Captures long-range correlations across the Raman shift axis.	Computationally heavy; vanishing gradients on broad spectra.	[70]
**Unsupervised learning models**				
PCA	Dim.Red. ^3^	Reduces dimensions and acts as a secondary noise filter.	Discards nonlinear interactions in complex biological matrices.	[46]
t-SNE	Dim.Red. ^3^	Excellent for 2D visualization of complex SERS clusters.	Not predictive; cannot map new unseen spectra to clusters.	[60]
K-Means	Clustering	Rapid blind grouping of unknown SERS mixtures.	Highly sensitive to baseline drift and cosmic ray spikes.	[61]
Hierarchical	Clustering	Reveals spectral similarities via dendrogram visualization.	Computationally heavy for large SERS mapping datasets.	[62]

^1^ Classification and Dimensionality Reduction; ^2^ Classification and Regression; ^3^ Dimensionality Reduction.

**Table 3:** Summary of ML-assisted SERS applications highlighting specific analytical challenges and methodological insights.

Application	SERS-Specific Challenge	ML Strategy & Algorithm	Key Advantage	Ref.
Illicit Drugs & Forensics	Severe spectral overlap among structurally similar analogs.	Nonlinear Classification (PCA-SVM, PCA-DA)	Resolves subtle spectral differences beyond human-resolvable peak assignments.	[75,76]
Complex Biospectra (DL)	High dimensionality and interdependent spectral features.	Hierarchical Feature Learning (2D-CNN)	Preserves inter-channel peak correlations without relying on manual feature engineering.	[77]
Quantitative Analysis	Nonlinear concentration-response and matrix interference.	Regression & Rule Extraction (RF, MCR-ALS)	Enables robust quantification; RF provides feature importance for chemical interpretability.	[78,79]
Trace Sequence Variations	Weak, sequentially distributed spectral signatures.	Sequence-Aware Modeling (LSTM, RNN)	Captures long-range spectral dependencies across adjacent Raman shifts.	[80]
Low-Abundance Biomarkers	Ultra-trace signals (picomolar level) buried in background noise.	Chemometric Regression	Improves analytical sensitivity, validating the presence of low-level targets (e.g., miRNA).	[81]
Metabolomic Biomarkers	High-dimensional, highly correlated global spectral variations.	Dim. Re & Class. (sPLS-DA & SVM)	Extracts global spectral patterns linked to disease states rather than isolated peaks.	[82]
Multiplex Profiling	Overlapping signals from multiple co-existing surface proteins.	Multivariate Modeling (PLS-DA)	Integrates multi-marker spectral features into a unified and robust diagnostic framework.	[83]
Multimodal Integration	Cross-platform variability and limited single-mode accuracy.	Data Fusion (PCA-SVM)	Synergistic integration of SERS and FTIR significantly enhances diagnostic discrimination.	[84]
Explainable AI (XAI)	“Black-box” nature of DL limits clinical trust and interpretability.	Feature Attribution (DNN & Score-CAM)	Attributes classification decisions to physically meaningful spectral peaks (“digital biomarkers”).	[85]

## Data Availability

No new data were created or analyzed in this study. Data sharing is not applicable to this article.

## Abbreviations

The following abbreviations are used in this manuscript:

SERS	Surface-enhanced Raman spectroscopy
ML	Machine learning
SPR	Surface plasmon resonance
AgNPs	Silver nanoparticles
AuNPs	Gold nanoparticles
LSPR	Localized surface plasmon resonance
CCD	Charge-coupled device
AsLS	Asymmetric least squares
ALS	Asymmetric least squares
SNR	Signal-to-noise ratio
COW	Correlation optimized warping
DTW	Dynamic time warping
GANs	Generative adversarial networks
PCA	Principal component analysis
LDA	Linear discriminant analysis
PLS-DA	Partial least squares discriminant analysis
SVM	Support vector machines
RF	Random forests
PLSR	Partial least squares regression
CNNs	Convolutional neural networks
AUC-ROC	Area under the receiver operating characteristic curve
MSE	Mean squared error
RMSE	Root mean squared error
MAE	Mean absolute error
DT	Decision trees
NB	Naïve Bayes
KNN	K-nearest neighbors
RT	Regression trees
t-SNE	t-distributed stochastic neighbor embedding
DL	Deep learning
ANNs	Artificial neural networks
MLP	Multi-layer perceptron
RNNs	Recurrent neural networks
LSTM	Long short-term memory
GRU	Gated recurrent unit
ResNet	Residual neural networks
DA	Discriminant analysis
MCR	Multivariate curve resolution
sPLS-DA	Sparse PLS discriminant analysis
ATR-FTIR	Attenuated total reflectance—Fourier transform infrared spectroscopy
XAI	Explainable artificial intelligence
DNN	Deep neural network
Score-CAM	Score-weighted visual explanations for convolutional neural networks
FDTD	Finite-difference time-domain
BP	Backpropagation
cVAE	conditional variational autoencoder
2D	Two-dimensional
TMDCs	Transition metal dichalcogenides
APN	Absorption prediction network
IDN	Inverse design network
E-field	Electric-field
PU	Periodic unit
CPUs	Central processing units
PINNs	Physical information neural networks
